# Laparoscopic Versus Robot-Assisted Versus Transanal Low Anterior Resection: 3-Year Oncologic Results for a Population-Based Cohort in Experienced Centers

**DOI:** 10.1245/s10434-021-10805-5

**Published:** 2021-10-04

**Authors:** T. A. Burghgraef, J. C. Hol, M. L. Rutgers, R. M. P. H. Crolla, A. A. W. van Geloven, R. Hompes, J. W. A. Leijtens, F. Polat, A. Pronk, A. B. Smits, J. B. Tuynman, E. G. G. Verdaasdonk, P. M. Verheijen, C. Sietses, E. C. J. Consten

**Affiliations:** 1 Department of Surgery, Meander Medical Center, Amersfoort, The Netherlands; 2 Department of Surgery, University Medical Center Groningen, Groningen, The Netherlands; 3 Department of Surgery, Hospital Gelderse Vallei, Ede, The Netherlands; 4 Department of Surgery, Amsterdam UMC, Locatie VUmc, Amsterdam, The Netherlands; 5 Department of Surgery, Amsterdam UMC, Locatie AMC, Amsterdam, The Netherlands; 6 Department of Surgery, Amphia Hospital, Breda, The Netherlands; 7 Department of Surgery, Tergooi Hospital, Hilversum, The Netherlands; 8 Department of Surgery, Laurentius Hospital, Roermond, The Netherlands; 9 Department of Surgery, Diakonessenhuis, Utrecht, The Netherlands; 10 Department of Surgery, St. Antonius Hospital, Nieuwegein, The Netherlands; 11 Department of Surgery, Jeroen Bosch Hospital, Den Bosch, The Netherlands; 12 Department of Surgery, Canisius Wilhelmina Hospital, Nijmegen, The Netherlands

## Abstract

**Background:**

Laparoscopic, robot-assisted, and transanal total mesorectal excision are the minimally invasive techniques used most for rectal cancer surgery. Because data regarding oncologic results are lacking, this study aimed to compare these three techniques while taking the learning curve into account.

**Methods:**

This retrospective population-based study cohort included all patients between 2015 and 2017 who underwent a low anterior resection at 11 dedicated centers that had completed the learning curve of the specific technique. The primary outcome was overall survival (OS) during a 3-year follow-up period. The secondary outcomes were 3-year disease-free survival (DFS) and 3-year local recurrence rate. Statistical analysis was performed using Cox-regression.

**Results:**

The 617 patients enrolled in the study included 252 who underwent a laparoscopic resection, 205 who underwent a robot-assisted resection, and 160 who underwent a transanal low anterior resection. The oncologic outcomes were equal between the three techniques. The 3-year OS rate was 90% for laparoscopic resection, 90.4% for robot-assisted resection, and 87.6% for transanal low anterior resection. The 3-year DFS rate was 77.8% for laparoscopic resection, 75.8% for robot-assisted resection, and 78.8% for transanal low anterior resection. The 3-year local recurrence rate was in 6.1% for laparoscopic resection, 6.4% for robot-assisted resection, and 5.7% for transanal procedures. Cox-regression did not show a significant difference between the techniques while taking confounders into account.

**Conclusion:**

The oncologic results during the 3-year follow-up were good and comparable between laparoscopic, robot-assisted, and transanal total mesorectal technique at experienced centers. These techniques can be performed safely in experienced hands.

The primary surgical treatment for rectal cancer is total mesorectal excision (TME).^1^ After the introduction of laparoscopic TME (L-TME), large randomized trials showed that oncologic outcomes for minimally invasive L-TME are not superior to open TME.^2–5^ However, because L-TME has led to an improvement in short-term outcomes such as hospital length of stay,^6^ it has become the standard technique in most Western countries.^7^

Despite its short-term benefits, laparoscopic surgery has not been proven superior to open surgery with regard to oncologic outcomes.^2–5^ Especially for distal tumors deep in the pelvis, the laparoscopic technique has technical limitations. To overcome these limitations, two new minimally invasive techniques have been introduced for the surgical resection of rectal carcinoma: robot-assisted TME (R-TME) and transanal TME (TaTME).

Adequate comparative studies investigating L-TME, R-TME, and TaTME are lacking. Most studies are single-center cohort series reporting on the comparison of only two techniques,^8^ whereas studies comparing all three minimally invasive techniques are scarce.^9^ Additionally, most studies did not take into account the learning curve of the new technique, which is associated with worse outcomes.^10,11^ Despite the limited number of comparative studies, results show equality of the three techniques with regard to short-term results.^8,11–13^

Evidence regarding oncologic outcomes is scarce. Lately, case series have reported on the oncologic results of minimally invasive techniques for TME. High local recurrence rates have been found in series reporting on the initial cases managed using TaTME, leading to the suspension of TaTME in Norway.^14,15^ Similarly, a high local recurrence rate has been reported in a comparative study of R-TME.^16^ On the other hand, low local recurrence rates for both techniques have been reported as well.^17–20^

In conclusion, robust data comparing all three techniques regarding oncologic outcomes taking into account the learning curve are lacking. Therefore, this multicenter cohort study aimed to compare the 3-year oncologic outcomes of laparoscopic, robot-assisted, and transanal sphincter-saving TME performed by surgeons well beyond their learning curve.

## Methods

A retrospective multicenter cohort study was performed to compare L-TME with R-TME and TaTME performed in five dedicated laparoscopy centers, three dedicated robot-assisted centers, and three dedicated TaTME centers between 2015 and 2017. A protocol regarding the design, methods, and statistical analysis was composed before initiation of the study. This study was reported in accordance with the Strengthening the Reporting of Observational Studies in Epidemiology (STROBE) guidelines.^21^

### Design

Centers were able to participate in this multicenter population-based cohort if they were “dedicated centers” for L-TME, R-TME, or TaTME and only one of the techniques was the standard technique. In addition, colorectal surgeons performing TME in the specific center had to be well beyond the learning curve for the specific technique, which is estimated to be about 40 procedures for R-TME and TaTME.^22–25^

The dedicated robot-assisted centers were three large teaching hospitals who started using R-TME in 2011, 2012, and 2014, respectively. The dedicated TaTME centers were three large teaching hospitals who started using TaTME in 2012, 2012, and 2014, respectively. With an average of 50 procedures per center annually and a maximum of two dedicated colorectal surgeons per center performing the procedure, it was estimated that all surgeons in the dedicated TaTME and robot-assisted centers that started using R-TME or TaTME in 2011 or 2012 were well beyond their learning curve at the beginning of the study. The two centers with start dates in 2014 fulfilled the learning curve in 2015. Therefore, in these centers, patients were included from 1 January 2016 until 31 December 2017. Finally, with more than 10 years of experience performing L-TME in the dedicated laparoscopic centers, these surgeons were estimated to be well beyond their learning curve as well. Altogether, 12 L-TME surgeons, 6 R-TME surgeons, and 6 TaTME surgeons participated in this study.

### Patients

Patients were eligible for inclusion if they had a diagnosis of rectal cancer according to the new definition using the sigmoidal take-off on magnetic resonance imaging (MRI) or computed tomography (CT),^26^ were older than 18 years, were registered in the prospective Dutch ColoRectal Audit (DCRA), and had undergone an L-TME in a dedicated laparoscopic center, an R-TME in a dedicated robot-assisted center, or a TaTME in a dedicated TaTME center. Patients were excluded if they had undergone surgery in an emergency setting, had a synchronous metastasis during diagnosis of rectal cancer, had undergone treatment with palliative intent, had more than one colorectal tumor at diagnosis, had undergone hyperthermic intraperitoneal chemotherapy (HIPEC) or intraoperative radiotherapy (IORT), had undergone transanal minimally invasive surgery (TAMIS), had undergone an abdominal perineal resection (APR), or had a surgeon performing the procedure who did not fulfil the learning curve. Each patient was discussed by a local multidisciplinary cancer board, and neoadjuvant treatment was offered according to the current Dutch National guidelines for colorectal cancer.^27^

### Outcomes

The primary outcome was overall survival (OS) after the 3-year follow-up period. Overall survival was defined as being alive at the 3-year follow-up evaluation. The secondary outcomes were disease-free survival (DFS) after the 3-year follow-up period, systemic recurrence after the 3-year follow-up period, local recurrence after the 3-year follow-up period, pattern of local recurrence, location of distant metastasis, and permanent stoma rate at the end of the follow-up period. Disease-free survival was defined as being alive without recurrent disease after the 3-year follow-up period. Systemic recurrence was defined as any distant metastasis, either pathologically proven or considered to be a lesion suspect for metastasis on imaging that showed growth on consecutive imaging. Local recurrence was defined as a tumor deposit located in the pelvic cavity, with pathologically proven adenocarcinoma or growth on consecutive imaging if histopathologic confirmation was absent. Multifocal local recurrence was defined as two or more separate deposits of recurrence in the pelvis. Location of local recurrence was classified according to the classification by Georgiou et al.^28^

The baseline characteristics were age, sex, body mass index (BMI), American Society of Anesthesiologists (ASA) classification, history of abdominal surgery, distance of the tumor from the anorectal junction (ARJ) on MRI, low defined rectal tumor according to the English National Low Rectal Cancer Development Programme (LOREC),^29^ mesorectal fascia involvement (MRF) on preoperative MRI, neoadjuvant (chemo)radiation therapy, preoperative tumor-node-metastasis (TNM) classification, and type of procedure (low anterior resection [LAR] with end colostomy or LAR with primary anastomosis). Furthermore, pathologic TNM classification, histologic tumor type, positive circumferential resection margin (≤ 1 mm), quality of the TME specimen according to Quirke,^30^ 30-day postoperative mortality, 30-day surgical complications graded according to the Clavien-Dindo classification,^31^ and anastomotic leakage rate at the end of the follow-up period according to the definition of the International Study Group of Rectal Cancer^32^ were registered.

### Data Sources

All the hospitals provided their local DCRA data, including the unique patient number. After pseudonymisation, missing and incomplete data were added in the database by accessing the electronical medical record (EMR) of the local hospitals. In addition, local recurrence, systemic recurrence, survival data, and follow-up data were added using the EMR of the local hospitals. All preoperative MRI data were reviewed by trained researchers. Informed consent was deemed unnecessary according to the Dutch Medical Treatment Agreement Act. The regional medical ethical committee and local ethical committees of all the hospitals gave approval for the study (MEC-U, AW19.023 W18.100).

### Statistical Methods

Categorical data are presented as number and percentages. Continuous variables are presented as mean and standard deviation or median and interquartile range (IQR) depending on the distribution. Survival curves of the patients were plotted in Kaplan-Meier graphs. To control for confounding factors that might have influenced choice of the surgical technique, a Cox regression using a backward model was performed comparing the three techniques for 3-year overall-survival, 3-year DFS, 3-year local recurrence, and 3-year systemic recurrence. For the Cox regressions, missing data were imputed using multiple imputation if the type of the missing data was missing at random or missing completely at random.

The variables used in the Cox regression were age (continuous), sex, BMI (continuous), history of abdominal surgery, ASA classification (1/3 vs 3/4), distance of the tumor to the ARJ on MRI in centimeters (continuous), neoadjuvant (chemo)radiation therapy, and a variable combining clinical T stage and MRF involvement on preoperative MRI. This variable was graded as cT3 without MRF involvement, cT3 with MRF involvement, cT4a or cT4b. Whereas cT4a was defined as a tumor invading in the ventral peritoneum, cT4b was defined as a tumor invading the sphincter complex or an adjacent organ.

The regression models were evaluated for assumptions and adjusted if necessary. Hazard ratios (HRs) and *p* values were used to interpret the results. A confidence interval either below or above 1 was interpreted as significant. Analyses were performed with R (version 3.6.1) using the “survival” and “survminer” packages.

## Results

The study identified 1834 patients as eligible between 1 January 2015 and 31 December 2017. After excluding 764 patients, the study had 1070 candidate patients. Of these patients, 487 had surgery in a dedicated laparoscopy center, 340 had surgery in a dedicated robot-assisted center, and 243 had surgery in a dedicated TaTME center. Additionally, 153 patients had a resection performed by a technique that was not the standard technique of the dedicated center, and 300 patients underwent an abdominoperineal resection (APR) and were therefore excluded from the study.

**Fig. 1:**
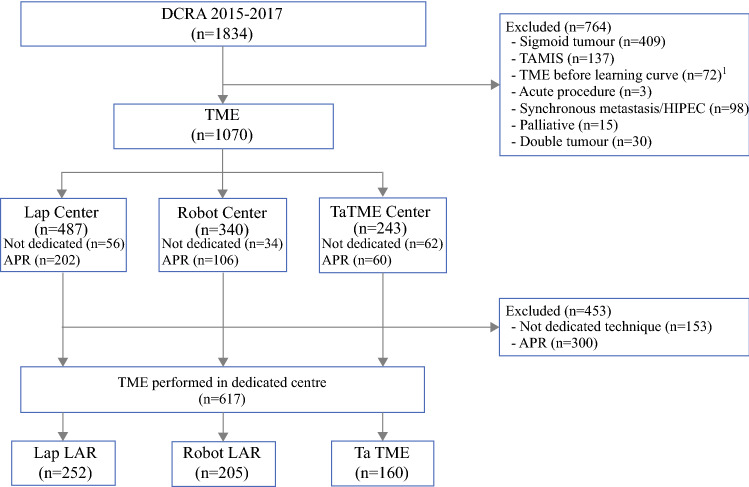
Flow diagram of patients included in the study. *DCRA* Dutch Colorectal Audit, *TME* total mesorectal excision, *LAR* low anterior resection, *Lap* laparoscopic, *Robot* robot-assisted, *TaTME* transanal TME, *HIPEC* hyperthermal intraperitoneal chemotherapy, *IORT* intraoperative radiotherapy, *TEM* transanal endoscopic microsurgery, *APR* abdominoperineal resection.^1^ Patients who underwent surgery in 2015 at a TaTME or robot-assisted center that started performing TaTME or robot-assisted TME respectively in 2014

Finally, 617 patients who underwent a low anterior resection (LAR) in a dedicated center were included in the analysis comprising 252 laparoscopic (L-LAR), 205 robot-assisted (R-LAR), and 160 TaTME procedures (Fig. 1). Abdominal perineal resection was performed for 202 patients (41.5%) in a laparoscopy center, for 106 patients (31.2%) in a robot-assisted center, and for 60 patients (24.7%) in a TaTME center. In the laparoscopy centers 56 (11.4%) patients did not undergo TME by the dedicated technique, and 27 (5.5%) of these patients underwent an open resection. In the robot-assisted and TaTME centers, respectively 34 (10.0%) and 62 (25.5%) patients did not undergo the dedicated technique, and respectively 5 (1.5%) and 8 (3.2%) of these patients underwent an open resection.

### Patient Characteristics

The mean patient age was higher in the laparoscopic L-LAR group than in the R-LAR and TaTME groups (68 ± 9.7 years vs. 66 ± 10.2 and 65 ± 10.9 years; *p* = 0.04). Data regarding race were not provided in the dataset. The median tumor distance from the ARJ on MRI was significantly greater in the L-LAR and R-LAR groups than in the TaTME group (7 ± 5.9 and 8 ± 6.9 cm vs. 4 ± 3.6 cm; *p* < 0.001). The L-LAR group had significantly less mesorectal fascia involvement than the R-LAR and TaTME groups (17.1% vs. 26.4% and 28.9%; *p* = 0.009). Furthermore, significantly fewer primary anastomoses were constructed in the L-LAR group than in the R- LAR and TaTME groups (68.3% vs. 91.2% and 82.5%; *p* < 0.001). Additionally, a significantly higher permanent stoma rate at the end of the follow-up period was observed in the L-TME group than in the R-TME and TaTME groups (42.1% vs. 22.0% and 31.2%) (Tables 1 and 2). Finally, the positive circumferential rate was 2.7% in the L-LAR group, 4.5% in the R-LAR group, and 3.2% in the TaTME group (*p* = 0.58).

**Table 1:** Baseline characteristics

		L-LAR	R-LAR	TaTME	
		(*n* = 252) *n* (%)	(*n* = 205) *n* (%)	(*n* = 160) *n* (%)	*p* Value
Mean age (years)		68 ± 9.7	66 ± 10.2	65 ± 10.9	0.04
Mean BMI (kg/m^2^)		26 ± 4.4	26 ± 3.8	26 ± 4.3	0.87
Sex	Male	155 (61.5)	128 (62.4)	111 (69.4)	0.24
	Female	97 (38.5)	77 (37.6)	49 (30.6)	
ASA classification	1	59 (23.4)	45 (22.0)	35 (21.9)	0.67
	2	137 (54.4)	123 (60.0)	95 (59.4)	
	3	53 (21.0)	37 (18.0)	29 (18.1)	
	4	3 (1.2)	0 (0.0)	1 (0.6)	
History of abdominal surgery		79 (31.3)	46 (22.4)	37 (23.1)	0.06
Median distance tumor to ARJ: cm (IQR)		7 (5–9)	8 (6–9)	4 [3, 6]	< 0.001
Low defined tumor^a^	Yes	110 (44.2)	69 (34.5)	80 (50.0)	0.01
	Missing	3 (1.2)	5 (2.4)	0 (0.0)	
Mesorectal fascia involvement on preoperative MRI	MRF+	43 (17.1)	53 (26.4)	46 (28.9)	0.009
	Missing	0 (0.0)	5 (2.4)	1 (0.6)	
cT	1	7 (2.8)	5 (2.4)	6 (3.8)	0.69
	2	80 (31.7)	66 (32.2)	42 (26.4)	
	3	156 (61.9)	117 (57.1)	104 (65.4)	
	4a	4 (1.6)	9 (4.3)	2 (1.3)	
	4b	5 (2.0)	7 (3.4)	5 (3.1)	
	Missing	0 (0.0)	1 (0.5)	1 (0.6)	
cN	0	108 (42.9)	87 (42.4)	86 (53.8)	0.04
	1	88 (34.9)	68 (33.2)	54 (33.8)	
	2	56 (22.2)	50 (24.4)	20 (12.5)	
	Missing	0 (0.0)	1 (0.5)	0 (0.0)	
Neoadjuvant therapy	None	109 (44.0)	82 (40.4)	64 (40.0)	0.46
	Radiotherapy	83 (33.5)	69 (34.0)	47 (29.4)	
	Chemoradiation	56 (22.6)	52 (25.6)	49 (30.6)	
	Missing	4 (1.6)	2 (1.0)	0 (0.0)	
Procedure	LAR + colostomy	80 (31.7)	18 (8.8)	28 (17.5)	< 0.001
	LAR + anastomosis	172 (68.3)	187 (91.2)	132 (82.5)	
Histologic type	Adenocarcinoma	240 (95.2)	196 (95.6)	155 (96.9)	0.38
	Mucinous	12 (4.8)	9 (4.4)	4 (2.5)	
	Other	0 (0.0)	0 (0.0)	1 (0.6)	
Differentiation	Well/moderate	233 (92.5)	184 (89.8)	146 (91.2)	0.90
	Poor	7 (2.8)	7 (3.4)	5 (3.1)	
	Unknown	12 (4.8)	14 (6.8)	9 (5.6)	
pT	0	15 (6.0)	14 (6.9)	15 (9.4)	0.49
	1	28 (11.2)	25 (12.3)	22 (13.8)	
	2	99 (39.4)	66 (32.4)	55 (34.6)	
	3	107 (42.6)	93 (45.6)	64 (40.3)	
	4	2 (0.8)	6 (2.9)	3 (1.9)	
	Missing	1 (0.4)	2 (1.0)	1 (0.6)	
pN	0	166 (65.9)	136 (66.7)	114 (71.2)	0.60
	1	61 (24.2)	47 (23.0)	28 (17.5)	
	2	25 (9.9)	21 (10.3)	18 (11.2)	
	Missing	0 (0.0)	1 (0.5)	1 (0.6)	
CRM^b^	(≤ 1 mm)	4 (1.7)	9 (4.7)	4 (2.8)	0.18
	Missing	1 (0.4)	2 (1.0)	1 (0.6)	
Incomplete TME specimen		7 (2.9)	9 (4.4)	2 (1.3)	0.23
	Missing	8 (3.2)	1 (0.5)	5 (3.1)	
30-Day surgical complications		83 (32.9)	82 (40.0)	49 (30.6)	0.15
	CD ≥ 3	53 (21.0)	43 (21.0)	40 (25.0)	0.58
Anastomotic leakage^c^		30 (11.9)	33 (16.0)	26 (16.2)	0.85
30-Day mortality		4 (1.6)	3 (1.5)	0 (0.0)	0.29

*L-LAR* laparoscopic low anterior resection, *R-LAR* robot-assisted low anterior resection, *TaTME* transanal total mesorectal excision, *BMI* body mass index, *ASA* American Society of Anesthesiologists, *ARJ* anorectal junction, *IQR* interquartile range, *MRI* magnetic resonance imaging, *MRF* mesorectal fascia involvement, *CRM* circumferential resection margin, *TME* total mesorectal excision, *CD* Clavien-Dindo classification grade

^1^Defined according to the English National Low Rectal Cancer Development Programme (LOREC)

^b^Positive CRM rate as percentage of patients with ypT1-4

^c^Anastomotic leakage as percentage of LAR with primary anastomosis

**Table 2:** Oncologic results not corrected for preoperative characteristics

		L-LAR	R-LAR	TaTME	
		(*n* = 252) *n* (%)	(*n* = 205) *n* (%)	(*n* = 160) *n* (%)	*p* Value
Median follow-up: months (IQR)		36 (25–46)	37 (26–45)	35 [25, 45]	0.83
3-Year overall survival		159 (90.0)	124 (90.4)	82 (87.6)	0.90
3-Year disease-free survival		121 (77.8)	97 (75.8)	73 (78.8)	0.76
3-Year local recurrence		12 (6.1)	12 (6.4)	7 (5.7)	0.82
	Anterior	0 (0.0)	1 (0.5)	0 (0.0)	
	Lateral	3 (1.1)	1 (0.5)	1 (0.6)	
	Inferior	5 (2.0)	2 (1.0)	0 (0.0)	
	Central anastomotic	2 (0.8)	5 (2.4)	3 (1.9)	
	Central non-anastomic	6 (2.4)	6 (2.9)	0 (0.0)	
	Peritoneal refletion	1 (0.4)	0 (0.0)	0 (0.0)	
	Multifocal recurrence	1 (7.1)	3 (18.8)	0 (0.0)	0.47
3-Year systemic recurrence		32 (15.1)	28 (15.9)	15 (10.1)	0.43
	Liver	21 (8.3)	13 (6.3)	8 (5.0)	
	Lung	17 (6.7)	14 (6.8)	8 (5.0)	
	Peritoneal	3 (1.2)	5 (2.4)	2 (1.2)	
	Bone	1 (0.4)	2 (1.0)	2 (1.2)	
	Ovary	1 (0.4)	0 (0.0)	0 (0.0)	
	Brain	1 (0.4)	0 (0.0)	0 (0.0)	
	Other	4 (1.6)	2 (1.0)	4 (2.5)	
Permanent stoma rate^a^		106 (42.1)	45 (22.0)	50 (31.2)	<0.001

*L-LAR* laparoscopic low anterior resection, *R-LAR* robot-assisted low anterior resection, *TaTME* transanal total mesorectal excision, *IQR* interquartile range

^a^Permanent stoma rate at the end of the follow-up period

### Overall Survival

**Fig. 2:**
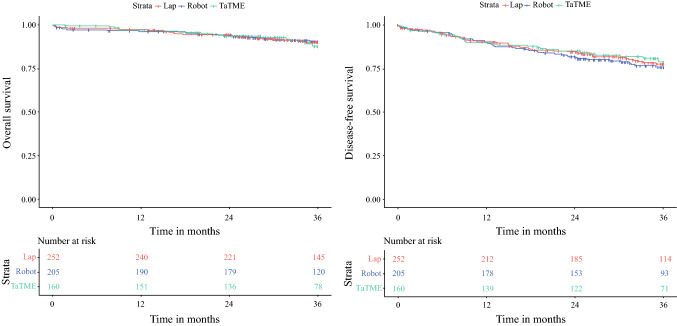
Curves showing 3-year overall and disease-free survival. *Lap* laparoscopic low anterior resection, *Robot* robot-assisted low anterior resection, *TaTME* transanal total mesorectal excision

The OS rate during the 3-year follow-up period was 90.0% in the L-LAR group, 90.4% in the R-LAR group, and 87.6% in the TaTME group (Table 2; Fig. 2). Cox regression did not show an association of the surgical technique with OS (Table 3). The factors associated with worse OS were age (HR 1.03; 95% confidence interval [CI], 1.00–1.06), ASA 3 and 4 (HR 6.63; 95% CI 3.66–12.0), cT3 MRF-tumor (HR, 2.05; 95% CI 1.01–4.16), and cT4b tumor (HR 6.77; 95% CI 2.04–22.4). Increased distance of the tumor to the ARJ was associated with improved OS (HR 0.88; 95% CI 0.79–0.98) (Table 3).

**Table 3:** Cox regression of 3-year overall survival (OS) and disease-free survival (DFS)

		3-Year OS			3-Year DFS		
		HR	95% CI	*p* Value	HR	95% CI	*p* Value
Approach	L-LAR	–	–	–	–	–	–
	R-LAR	1.31	(0.69–2.50)	0.42	1.18	(0.78–1.79)	0.44
	TaTME	0.78	(0.37–1.63)	0.50	0.75	(0.45–1.28)	0.29
Age		1.03	(1.00–1.06)	0.05	1.00	(0.99–1.02)	0.65
BMI (kg/m^2^)		0.99	(0.94–1.05)	0.76	0.98	(0.94–1.02)	0.32
Sex	Male	1.10	(0.59–2.03)	0.75	1.33	(0.89–1.99)	0.16
ASA classification	3/4	6.62	(3.66–12.0)	< 0.001	2.82	(1.86–4.28)	<0.001
History of abdominal surgery	Yes	0.93	(0.51–1.72)	0.83	1.26	(0.83–1.91)	0.27
Distance tumor to ARJ		0.88	(0.79–0.98)	0.02	0.94	(0.88–1.02)	0.12
cT/MRF	cT3, MRF-	2.05	(1.01–4.16)	0.05	1.76	(1.07–2.90)	0.03
	cT3, MRF+	0.84	(0.31–2.32)	0.74	1.23	(0.64–2.35)	0.53
	cT4a	1.53	(0.31–7.42)	0.60	3.16	(1.23–8.14)	0.02
	cT4b	6.77	(2.04–22.4)	0.001	7.89	(3.62–17.2)	< 0.001
cN	cN+	0.91	(0.39–2.12)	0.83	0.84	(0.49–1.44)	0.53
Neoadjuvant therapy	Yes	1.11	(0.45–2.73)	0.83	1.25	(0.69–2.26)	0.46

*HR* hazard ratio, *CI* confidence interval, *L-LAR* laparoscopic low anterior resection, *R-LAR* robot-assisted low anterior resection, *TaTME* transanal total mesorectal excision, *BMI* body mass index, *OR* odds ratio, *BMI* body mass index, *ASA* American society of anesthesiologists, *ARJ* anorectal junction

### Disease-Free Survival

The DFS rate during the 3-year follow-up period was 77.8% in the L-LAR group, 75.8% in the R-LAR group, and 78.8% in the TaTME group (Table 2; Fig. 2). Cox regression did not show an association of the surgical technique with DFS. The factors associated with worse DFS were ASA 3 and 4 (HR 2.82; 95% CI 1.86–4.28), cT3 MRF-tumor (HR 1.76; 95% CI 1.07–2.90), cT4a tumor (HR 3.16; 95% CI 1.23–8.14), and cT4b tumor (HR 7.89; 95% CI 3.62–17.2) (Table 2).

### Local Recurrence

The local recurrence rate was 6.1% in the L-LAR group, 6.4% in the R-LAR group, and 5.7% in the TaTME group during the 3-year follow-up period. Multifocal recurrence was seen in 1 (7.1%) of 12 laparoscopic patients, 3 (18.8%) of 13 robot-assisted patients, and none of the TaTME patients (Table 2). Cox regression did not show an association of the surgical technique with local recurrence. The factors associated with local recurrence at 3 years were cT4a tumor (HR 11.58; 95% CI 2.40–55.8) and cT4b tumor (HR 12.94; 95% CI 2.64–64.0) (Table 4).

**Table 4:** Cox regression of 3-year local recurrence and 3-year systemic recurrence

		3-year Local recurrence			3-year Systemic recurrence		
		HR	95% CI	*p* value	HR	95% CI	*p* value
Approach	L-LAR	–	–	–	–	–	–
	R-LAR	1.25	(0.54; 2.86)	0.60	1.03	(0.61; 1.73)	0.91
	TaTME	0.51	(0.17; 1.51)	0.23	0.74	(0.37; 1.49)	0.40
Age		1.00	(0.96; 1.03)	0.81	0.99	(0.97; 1.01)	0.34
BMI (kg/m2)		1.03	(0.95; 1.12)	0.46	1.02	(0.96; 1.08)	0.57
Sex	Male	1.74	(0.75; 4.06)	0.20	1.29	(0.78; 2.13)	0.32
ASA classification	3/4	1.98	(0.79; 4.95)	0.15	1.48	(0.81; 2.71)	0.20
History of abdominal surgery	Yes	1.04	(0.43; 2.53)	0.92	1.81	(1.08; 3.02)	0.02
Distance tumour to ARJ		0.88	(0.76; 1.02)	0.08	1.01	(0.93; 1.11)	0.76
cT/MRF	cT3, MRF-	2.24	(0.79; 6.33)	0.13	1.52	(0.78; 2.93)	0.22
	cT3, MRF+	2.24	(0.58; 8.57)	0.24	1.42	(0.62; 3.19)	0.40
	cT4a	11.58	(2.40; 55.8)	0.002	4.63	(1.55; 13.9)	0.006
	cT4b	12.94	(2.62; 64.0)	0.002	7.76	(2.82; 21.4)	< 0.001
cN	cN+	0.55	(0.20; 1.54)	0.26	0.98	(0.48; 2.00)	0.96
Neoadjuvant therapy	Yes	0.91	(0.30; 2.75)	0.87	1.56	(0.70; 3.48)	0.28

*TaTME* Transanal total mesorectal excision, *OR* odds ratio, *CI* confidence interval, *BMI* body mass index, *ASA* American society of anesthesiologists, *ARJ* anorectal junction

## Discussion

This study compared 3-year oncologic outcomes between L-LAR, R-LAR, and TaTME in dedicated centers while taking the learning curve into account. The results from this study showed equal oncologic outcomes for all three minimally invasive techniques. Comparable OS, DFS, local recurrence, and systemic recurrence were observed during the 3-year follow-up period. To our knowledge this is the first study to compare all three minimally invasive techniques performed by surgeons well beyond the learning curve of each specific technique, with the longest follow-up data presented to date.

The OS survival rates at 3 years in this study were 90.0% for the laparoscopic, 90.4% for the robot-assisted, and 87.6% for the TaTME technique. The corresponding DFS rates at 3 years were 77.8%, 75.8 and 78.8%. For both outcomes, no difference between the three techniques was observed in the multivariable Cox regression. First, these results showed the high quality of oncologic outcomes in the dedicated centers, underscoring our assumption that the included centers were dedicated and beyond the learning curve for the specific technique. The aforementioned rates are comparable with those of large trials comparing L-TME with open TME such as the AlaCaRT, ACOSOG Z6501, COREAN and COLOR II trials.^2,4,5,33^ All these trials used strict inclusion criteria and excluded ASA 4 patients or cT4 tumors. In contrast, the current population-based cohort presents a more realistic image of clinical practice, with better external validity than the randomized clinical trials.

Second, these results show comparable oncologic outcomes among all three techniques. This is the first analysis to compare all three techniques. To date, no comparative oncologic data regarding TaTME have been published. Retrospective cohort analyses regarding TaTME show a similar OS rate.^19,20^ Studies comparing oncologic results after R-TME with L-TME are scarce, but mainly confirm our results. Although studies show comparable OS and DFS between R-TME and L-TME.^16,34–36^ a recent propensity score-matched analysis showed significantly better OS and DFS in the R-TME group than in the L-TME group.^37^ However, this might have been caused by a relatively high rate of distant metastasis in the L-TME group, whereas the local recurrence rate was equal. Because systemic recurrence is suggested to be a mere result of the biologic behavior and tumor stage at presentation and a less relevant outcome regarding quality of surgery, the difference in OS and DFS might not be attributable to a difference in technique.

Local recurrence was present in 6.1% of L-LAR, 6.4% of R-LAR, and 5.7% of TaTME procedures. The multivariable Cox regression did not show any difference between the three techniques, indicating adequate surgical quality and safe surgery for all three minimally invasive techniques in the dedicated centers. These results are comparable with those of large randomized controlled trials comparing L-TME with open TME surgery. However, these trials did not include patients with T4 or T3 tumors that had mesorectal fascia involvement.^2,4,5,33^ Furthermore, we used the rectal cancer definition as proposed by D’Souza et al.^26^ The exclusion of “rectosigmoid”’ cancers could have led to the inclusion of relatively more low rectal cancers, and therefore to more difficult tumors because this is a known risk factor for local recurrence.^38^

Recently, local recurrence rates after TaTME in Norway were reported to be 9.5%, and a significant proportion of multifocal recurrences were reported, leading to a nationwide halt of TaTME.^14^ Similar results were seen in the initial cases of centers learning the TaTME technique in the Netherlands.^15^ However, higher local recurrence rates also have been reported in the initial cases of R-TME and L-TME.^16,39^ Although these studies suggest higher local recurrence rates during the learning curve, our results showed that adequate oncologic results can be obtained for L-LAR, R-LAR, and TaTME in experienced centers after fulfilment of the learning curve, in accordance with other series.^19,20,40^ Furthermore, no increased rate of multifocal recurrences was observed. Earlier reports on local recurrence after R-TME describe lower rates, but these retrospective cohorts had short follow-up times, with younger patients, lower BMI, and lower rates of neoadjuvant therapy than our cohort, which may suggest selection bias in these studies.^18,35,37,41–43^

Certain limitations of this study should be taken into account. First, this was a retrospective cohort study. Therefore, a certain degree of bias was present. However, we tried to overcome confounding by indication, using multivariable analysis to control for baseline characteristics that might have influenced the choice for a certain surgical technique preoperatively. Our primary aim was to assess whether surgical technique would influence oncologic outcomes for TME. Therefore, we took into account only preoperative variables and did not control for postoperative variables such as pathologic TNM stage or positive circumferential resection margins because these postoperative variables are a result of the surgical technique.

Preferably, a prospective randomized controlled trial should be performed to evaluate the three minimally invasive procedures. In practice, however, randomization is hard to achieve because it can be doubted whether surgeons could be equally trained in each technique. Therefore, this population-based cohort was possibly a suitable alternative providing the current state of surgical practice with high external validity, in contrast to randomized controlled trials showing mostly low external validity due to strict inclusion and exclusion criteria. Nevertheless, because this was a retrospective cohort, the results should be replicated in a prospective study.

Second, because the surgical techniques were performed in dedicated centers, the institution itself could have influenced the outcomes as well. Adjustments could not be made for culture-, surgeon-, or team-related factors. However, by including more than one center per group, we tried to reduce this effect.

Third, we chose to select only patients who underwent a TME and excluded patients who underwent an APR. The patients who required an APR in a dedicated TaTME center underwent either a laparoscopic or an open APR because an APR is not an indication for the TaTME technique in the current Dutch clinical practice. Because we were interested in comparing the robot-assisted technique with the laparoscopic and TaTME techniques, in order to create homogeneous groups we decided to exclude patients who needed an APR. However, because APR is associated with worse oncologic outcomes, this might have influenced outcomes. Nevertheless, by excluding APR in all three groups, we tried to reduce confounding.

Finally, although we included only patients who underwent a minimally invasive TME at a dedicated center in which the learning curve had been fulfilled, the difference in experience could not be reduced to nil. The 10-year experience of the laparoscopic surgeons still exceeded the 3- to 5-year experience of the robot-assisted and TaTME surgeons.

Despite these limitations, this is the first study to show good and comparable oncologic results between R-LAR , L-LAR, and TaTME in centers with profound experience using the specific technique. All three techniques showed adequate OS and DFS rates. Moreover, the recurrence rates are equal between the three minimally invasive techniques when performed by experienced surgeons, and multifocal recurrence rates are low. Therefore, oncologic safety can be achieved with all three minimally invasive techniques when performed by experienced surgeons. Prospective cohort studies comparing oncologic outcomes after fulfillment of the learning curve are needed to confirm our results.
